# Analysis of Postapproval Clinical Trials of Therapeutics Approved by the US Food and Drug Administration Without Clinical Postmarketing Requirements or Commitments

**DOI:** 10.1001/jamanetworkopen.2019.3410

**Published:** 2019-05-10

**Authors:** Joshua J. Skydel, Anita T. Luxkaranayagam, Sanket S. Dhruva, Joseph S. Ross, Joshua D. Wallach

**Affiliations:** 1 Tufts University School of Medicine, Boston, Massachusetts; 2 University of Connecticut, Storrs; 3 Department of Medicine, UCSF School of Medicine, University of California, San Francisco; 4 Section of Cardiology, San Francisco Veterans Affairs Health Care System, San Francisco, California; 5 Section of General Internal Medicine, Department of Internal Medicine, Yale School of Medicine, New Haven, Connecticut; 6 Department of Health Policy and Management, Yale University School of Public Health, New Haven, Connecticut; 7 Center for Outcomes Research and Evaluation, Yale-New Haven Hospital, New Haven, Connecticut; 8 Department of Environmental Health Sciences, Yale School of Public Health, New Haven, Connecticut; 9 Collaboration for Research Integrity and Transparency, Yale Law School, New Haven, Connecticut

## Abstract

**Importance:**

The US Food and Drug Administration (FDA) can use postmarketing requirements to mandate pharmaceutical companies to conduct clinical trials after the approval of novel therapeutics. Pharmaceutical companies can also agree to conduct nonmandated clinical trials as postmarketing commitments. However, when therapeutics are approved by the FDA without postmarketing requirements or postmarketing commitments, it is not well known how often pharmaceutical companies voluntarily conduct trials and report results monitoring safety or efficacy after approval.

**Objective:**

To characterize postapproval clinical trials sponsored by pharmaceutical companies of therapeutics initially approved by the FDA without clinical postmarketing requirements or commitments.

**Design, Setting, and Participants:**

This cross-sectional analysis included postapproval clinical trials conducted with at least 1 site in the United States sponsored by pharmaceutical companies of therapeutics first approved by the FDA from 2009 through 2012. Analyses were conducted June 11, 2018, to November 30, 2018.

**Main Outcomes and Measures:**

Postapproval clinical trials registered on ClinicalTrials.gov generating safety or efficacy data, characteristics including whether trials focused on approved or unapproved indications, study design elements, and rates of study completion and results reporting.

**Results:**

From 2009 through 2012, the FDA approved 110 novel therapeutics for 120 indications, of which 37 novel therapeutics for 39 indications did not have postmarketing requirements or commitments for new clinical studies at the time of first approval. For 31 therapeutics (83.8%), there were 600 postapproval clinical trials sponsored by pharmaceutical companies. Most trials investigated therapeutics for new indications (363 [60.5%]) or expanded populations of the originally indicated disease (122 [20.3%]). Trials were often small (median [interquartile range] enrollment, 44 [21-131] participants), nonrandomized (359 [59.8%]), unblinded (455 [75.8%]), and lacked comparators (381 [63.5%]). Approximately half of the trials (311 [51.8%]) assessed at least 1 clinical outcome. Of 300 terminated or completed trials, 204 trials (68.0%) had reported results on ClinicalTrials.gov a median (interquartile range) 16 (13-25) months after their primary completion date. For the 96 trials (32.0%) without reported results, a median (interquartile range) 35 (13-62) months had passed since their primary completion date.

**Conclusions and Relevance:**

Pharmaceutical companies frequently conducted clinical trials after approval, even when there were no clinical postmarketing requirements or commitments at approval. However, most of these trials evaluated new indications or expanded patient populations rather than monitored approved uses, and nearly half of the trials remained incomplete more than 5 years after original therapeutic approval.

## Key Points

**Question:**

When therapeutics are approved by the US Food and Drug Administration (FDA) without postmarketing requirements or commitments for clinical studies, how often do pharmaceutical companies voluntarily conduct trials and report results monitoring safety or efficacy after approval?

**Findings:**

In this cross-sectional study of 37 therapeutics approved by the FDA from 2009 through 2012 without postmarketing requirements or commitments for clinical studies, 31 had at least 1 postapproval trial generating safety or efficacy data sponsored by pharmaceutical companies and registered on ClinicalTrials.gov. Among 600 trials, only 12.0% exclusively evaluated the originally approved indication.

**Meaning:**

Pharmaceutical companies and associated sponsors conducted postapproval trials generating safety or efficacy data on most new therapeutics but most commonly for new or expanded indications, suggesting that the FDA may need to impose additional postmarketing requirements to generate further evidence for the original indications.

## Teaser

This cross-sectional study characterizes postapproval clinical trials sponsored by pharmaceutical companies of therapeutics approved by the US Food and Drug Administration (FDA) without postmarketing requirements or commitments.

## Introduction

The US Food and Drug Administration (FDA) traditionally requires pharmaceutical companies to demonstrate the safety and efficacy of novel therapeutics, generally based on at least 2 adequate and well-controlled trials, prior to obtaining marketing approval in the United States.^1,2^ However, over the last decade, the FDA has increasingly shifted toward life-cycle evaluation of drugs and biologics, placing greater emphasis on postmarket evidence generation as part of therapeutic evaluation.^1,3^ At the same time, FDA approval pathways aimed at expediting promising new drug approvals have been increasingly used by pharmaceutical companies.^2,3,4,5,6^ These pathways often enable approvals to be based on fewer and shorter clinical trials, which may not focus on clinical end points.^7^ Studies suggest that faster drug approvals are associated with higher rates of postmarket safety events,^8,9^ and boxed warnings, commonly referred to as *black box warnings*, are often added or updated in the postmarket period.^10^ Therefore, postapproval studies are essential for addressing uncertainties about therapeutic effectiveness and safety that remain after approval.^5,7,8,9,10^

Currently, the FDA can facilitate postapproval evidence generation by outlining postmarketing requirements. Postmarketing requirements are issued under 4 authorities, including the Food and Drug Administration Amendments Act (FDAAA), and must be conducted or completed by pharmaceutical companies to achieve specific research objectives, such as demonstrating clinical benefit for therapeutics receiving accelerated approval, completing pediatric studies, or assessing serious risks related to therapeutic use.^11,12^ Despite nearly 90% of novel therapeutics having postmarketing requirements, only 31% are for prospective cohort studies, registries, or clinical trials, the study designs most likely to generate clinical information relevant to physicians and patients.^13^ Pharmaceutical companies and the FDA can also formulate postmarketing commitments, which are agreed-on studies and trials not specifically required by the FDAAA or other regulations. However, approximately 45% of new therapeutics are approved without postmarketing commitments, and most of those agreed on are for nonclinical purposes, including chemistry, manufacturing, and controls studies.^14^ Furthermore, when new therapeutics are approved on the basis of limited evidence, such as trials focused on surrogate markers,^7^ confirmatory studies often lag years after approval, do not evaluate clinical outcomes, and fail to confirm benefit for approved indications.^15,16,17,18,19^

Pharmaceutical companies also voluntarily conduct postapproval trials to satisfy a variety of goals, including monitoring safety and effectiveness, educating clinicians, and expanding indications.^20,21^ However, when therapeutics are first approved without any formal postmarketing requirements or commitments for clinical studies, both of which are subject to FDA reporting requirements,^12,13,14,22,23^ it is unknown whether pharmaceutical companies continue to monitor approved indications or investigate additional uses for those products. Clinical trials conducted after approval can generate evidence about the harms, benefits, and optimal uses of novel therapeutics in patient populations that are larger and more diverse than those studied prior to approval. Considering this real-world usage, clinicians, patients, and other stakeholders stand to benefit from data that are responsive to developing clinical practices and that can inform decisions about on-label and off-label uses of currently marketed therapeutics. Opportunities may also exist to align the objectives of studies with the needs of clinicians and the FDA for data on risks and benefits that can guide clinical and regulatory decisions. Therefore, we characterized postapproval clinical trials for drugs and biologics initially approved without postmarketing requirements or commitments for new clinical studies. We assessed research objectives, study design characteristics, and rates of completion and reporting on ClinicalTrials.gov, focusing on clinical trials sponsored by pharmaceutical companies that enrolled patients at US study sites and generated safety or efficacy data.

## Methods

### Ethical Review and Reporting Guideline

This study was based on publicly available data that did not include patient contact or medical record review and therefore did not require institutional review board approval or informed consent. This study follows the Strengthening the Reporting of Observational Studies in Epidemiology (STROBE) reporting guideline for cross-sectional studies.

### Study Design and Sample

Using the Drugs@FDA database,^24^ 2 of us (J.J.S. and J.D.W.) identified all new therapeutics (ie, drugs and biologics) approved by the FDA from January 1, 2009, through December 31, 2012, without postmarketing requirements or commitments for new clinical studies (ie, clinical trials, prospective cohort studies, and registries), as described in previous work (eAppendix 1 and eTable 1 in the Supplement).^13^ The cutoff date was chosen to allow longer than 5 years for new clinical studies of therapeutics to be completed, a process that can include the registering, planning, and conducting of trials and analyzing and reporting of results.^13^ Generic drugs, reformulations, and new combinations of previously approved drugs were excluded.^7,13^ Approval letters and other documents in the Drugs@FDA database were used to identify the license holder for each therapeutic and whether the therapeutic underwent priority review, received accelerated approval pathway designation, and/or received an orphan drug designation.^7,13^ The first approved indication(s) for each therapeutic was classified according to the World Health Organization anatomic therapeutic classification system^25^ and collapsed into 4 categories: cancer and hematology; cardiovascular and diabetes; autoimmune, musculoskeletal, and dermatology; and other.

### Identification of Postapproval Trials on ClinicalTrials.gov

For novel therapeutics approved without postmarketing requirements or commitments for new clinical studies, ClinicalTrials.gov was used to identify registered clinical trials conducted by phamaceutical companies, their subsidiaries or parent organizations, or other developing and marketing partners in US study populations to generate clinical safety or efficacy evidence.^20,26,27^ ClinicalTrials.gov was chosen as the source of clinical trial data because the FDAAA requires the registration and reporting of results for controlled (ie, non–phase 1) clinical studies of any FDA-regulated drug or biologic product for any disease or condition.^28^

One of us (J.J.S.) entered the generic and brand names of each novel therapeutic into the intervention/treatment field of the advanced search feature of ClinicalTrials.gov (eAppendix 2 in the Supplement), and searches were filtered by restricting funder type to industry. The study start filter was set to 1 year prior to the therapeutic’s initial FDA approval date to locate ongoing studies that had not been completed or reported results prior to FDA approval. All ClinicalTrials.gov data were downloaded on July 10, 2018. One of us (J.D.W.) validated ClinicalTrials.gov search results in a 10% random sample of therapeutics with perfect concordance.

Two of us (J.J.S. and A.T.L.) reviewed the downloaded study entries and excluded trials that were completed prior to the FDA approval date; evaluated alternative FDA-approved or FDA-unapproved formulations of the active ingredient; provided therapeutics of interest at the study investigator’s discretion (eg, physician’s choice of mammalian target of rapamycin inhibitor); were observational; were trial extensions, rollover studies, follow-up studies, or substudies not enrolling new participants; were expanded access studies; had no study sites in the United States; enrolled healthy participants only; or did not evaluate any safety or efficacy end points (eAppendix 2 in the Supplement). Clinical trials were also excluded by 1 of us (J.J.S.) if the study’s sponsors did not include the therapeutic manufacturer, a subsidiary, or a company involved in the development or marketing of the therapeutic (eg, a licensed comarketer). When a trial assessed more than 1 therapeutic in our sample, resulting in duplicate study entries, 1 entry was chosen at random for inclusion (eTable 2 in the Supplement). Uncertainties were discussed among investigators (J.J.S., J.S.R., and J.D.W.) and resolved by consensus.

### Trial Data Abstraction

For each clinical trial registration, 2 of us (J.J.S. and A.T.L.) abstracted use of randomization; whether blinding was open label, single-blinded, or double-blinded or greater; whether a placebo, active comparator, or no comparator was used; enrollment; most recent status provided on ClinicalTrials.gov; whether results were reported on ClinicalTrials.gov; and dates of first submission, study start, primary completion, and summary results reported (eAppendix 3 in the Supplement). One of us (J.J.S.) used study title, description, and enrollment criteria to determine indications investigated by each trial. Indications for postapproval clinical trials were compared with the language used by the FDA when approving the original indication and classified as first FDA-approved indications, modified first FDA-approved indications, or FDA-unapproved indications (eTable 3 in the Supplement). Trials of modified first FDA–approved indications evaluated expanded patient populations for the same disease as the original indication, whereas FDA-unapproved indications referred to diseases for which therapeutics were not indicated at the time of first approval. We also determined whether postapproval trials evaluated supplemental FDA-approved indications, as outlined in supplemental new drug applications or other postapproval communications found in the Drugs@FDA database.

The primary, secondary, and any additional outcome measures listed for each clinical trial were classified by 1 of us (J.J.S.) based on whether they generated efficacy or safety evidence (eAppendix 3 in the Supplement).^7,13,17^ When multiple efficacy outcomes were evaluated, the highest level of evidence was recorded (from highest to lowest: clinical outcomes, clinical scales, and surrogate markers). Trials were classified as having other primary end points if they included pharmacokinetic or pharmacodynamic, drug-drug interaction, or maximum tolerated dose or dose-limiting toxicity measures that were assessed in addition to secondary safety or efficacy end points. One of us (J.D.W.) validated study abstractions in a 10% random sample with perfect concordance. Conflicts were resolved by consensus among investigators (J.J.S., J.S.R., and J.D.W.). Analyses were conducted from June 11, 2018, to November 30, 2019.

### Statistical Analysis

We used descriptive statistics to characterize novel therapeutics approved without clinical postmarketing requirements or commitments and postapproval trials of those therapeutics. The number of trials, investigated indications, study design elements, and completion and reporting rates were summarized for each therapeutic. We also calculated the time from study start to primary completion (ie, approximate study duration), time from FDA approval and from study start to results reporting (eAppendix 3 in the Supplement), time since study completion for completed or terminated trials without results reported (as of July 2018), and time from primary completion for trials without reported results (as of July 2018). We generated Kaplan-Meier plots estimating the time to first results reported on ClinicalTrials.gov. Analyses were performed using the survival and rms packages in R statistical software version 3.5.1 (The R Project for Statistical Computing).

## Results

### Characteristics of New Therapeutics

From 2009 through 2012, the FDA approved 110 new drugs and biologics for 120 indications. There were 37 therapeutics (33.6%) for 39 indications first approved without postmarketing requirements or postmarketing commitments for new clinical studies (Table 1). Therapeutics first approved without clinical postmarketing requirements or commitments included widely prescribed therapeutics (eg, apixaban, first approved for reduction of risk of stroke and systemic embolism caused by nonvalvular atrial fibrillation) and therapeutics with orphan designations for rare disease subtypes (eg, ivacaftor, first approved for patients with cystic fibrosis with a G551D mutation in the cystic fibrosis transmembrane conductance regulator gene) (eTable 1 in the Supplement). There were 31 drugs (83.8%) and 6 biologics (16.2%) in the final sample. The 14 therapeutics (37.8%) indicated for the treatment of cancer and hematologic disorders represented the largest therapeutic area. More than one-third of therapeutics (14 [37.8%]) received priority review, 3 (8.1%) were approved through the accelerated approval pathway, and 15 (40.5%) were designated as orphan drugs. The 3 novel therapeutics approved through the accelerated approval pathway had postmarketing requirements calling for completion and submission of results from ongoing trials rather than outlining new postapproval clinical studies.

**Table 1.** Characteristics of 37 New Therapeutics Approved Without Postmarketing Requirements or Postmarketing Commitments for New Clinical Studies by the US Food and Drug Administration, 2009-2012

Characteristic	No. (%)
Year of approval	
2009	10 (27)
2010	8 (22)
2011	8 (22)
2012	11 (30)
Class	
Drug	31 (84)
Biologic	6 (16)
Therapeutic area	
Cancer and hematology	14 (38)
Cardiovascular, diabetes, and hyperlipidemia	6 (16)
Autoimmune, musculoskeletal, and dermatology	5 (14)
Other	12 (32)
Priority review	
Yes	14 (38)
No	23 (62)
Accelerated approval	
Yes^a^	3 (8)
No	34 (92)
Orphan drug designation	
Yes	15 (41)
No	22 (60)

^a^ Each of the therapeutics that received accelerated approval (ofatumumab, ponatinib, and crizotinib) had postmarketing requirements for the completion and submission of results from ongoing prospective cohort studies or trials rather than requiring new prospective clinical studies.

### Postapproval Clinical Trials for New Therapeutics

**Figure 1.**
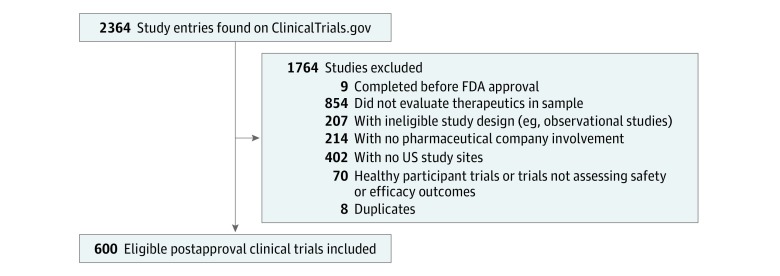
Flow Diagram of Postapproval Clinical Trials Included in Analysis FDA indicates US Food and Drug Administration.

Among the 37 therapeutics, 31 (83.8%) had at least 1 postapproval clinical trial registered on ClinicalTrials.gov that was conducted by pharmaceutical companies, which include approved manufacturers and associated collaborators, had at least 1 US study site, and assessed safety or efficacy end points. A total of 600 unique clinical trials were identified (Figure 1). The median (interquartile range [IQR]) number of postapproval clinical trials per therapeutic was 9 (3-19). Nearly three-quarters of trials of studied therapeutics (437 [72.8%]) were indicated for the treatment of cancer and hematologic disorders. Although most trials were for drugs (503 [83.8%]), the median (IQR) number of trials per therapeutic was greater for biologics (14 [4-23]) than for drugs (8 [3-19]). The median (IQR) time from first FDA approval to analysis for this study was 86 (77-104) months.

Most of the postapproval clinical trials (363 [60.5%]) studied new indications in diseases not included in the original FDA approval letter (Table 2). For 14 of 37 therapeutics (37.8%), no trials exclusively studied the first FDA-approved indication. There were 72 trials (12.0%) that exclusively studied the first FDA-approved indication, with enrollment requirements matching the approval language used by the FDA. A further 122 trials (20.3%) exclusively studied the first indicated disease but enrolled participants beyond the initially indicated population, such as pediatric populations or treatment-naive populations if the therapeutic was initially approved for previously treated patients. Multiple indications were studied in 43 trials (7.2%), including trials enrolling from multiple disease populations, and 22 of these trials (3.7%) included patients matching the first FDA-approved indication. There were 69 trials (11.5%) that evaluated indications that had received supplementary FDA approval as of July 2018.

**Table 2.** Indications Investigated for Therapeutics Approved Without Postmarketing Requirements or Postmarketing Commitments for New Clinical Studies by the FDA, 2009-2012

Therapeutic Characteristic	No. of Therapeutics	No. of Trials	No. (%)				
			Study Indication^a^				Supplemental FDA-Approved Indication^b^
			First FDA-Approved	Modified First FDA-Approved	FDA-Unapproved	Multiple	
Total	37	600	72 (12.0)	122 (20.3)	363 (60.5)	43 (7.2)	69 (11.5)
Class							
Drug	31	503	67 (13.3)	101 (20.1)	304 (60.4)	31 (6.2)	57 (11.3)
Biologic	6	97	5 (5.2)	21 (21.6)	59 (60.8)	12 (12.4)	12 (12.4)
Therapeutic area							
Cancer and hematology	14	437	26 (5.9)	94 (21.5)	280 (64.1)	37 (8.5)	47 (10.8)
Cardiovascular, diabetes, and hyperlipidemia	6	62	17 (27.4)	9 (14.5)	33 (53.2)	3 (4.8)	5 (8.1)
Autoimmune, musculoskeletal, and dermatology	5	33	14 (42.4)	3 (9.1)	16 (48.5)	0	3 (9.1)
Other	12	68	15 (22.1)	16 (23.5)	34 (50.0)	3 (4.4)	14 (20.6)
Priority review							
Yes	14	352	29 (8.2)	90 (25.6)	215 (61.1)	18 (5.1)	43 (12.2)
No	23	248	43 (17.3)	32 (12.9)	148 (59.7)	25 (10.1)	26 (10.5)
Accelerated approval							
Yes	3	63	4 (6.3)	16 (25.4)	30 (47.6)	13 (20.6)	4 (6.3)
No	34	537	68 (12.7)	106 (19.7)	333 (62.0)	30 (5.6)	65 (12.1)
Orphan status							
Yes	15	200	19 (9.5)	39 (19.5)	117 (58.5)	25 (12.5)	29 (14.5)
No	22	400	53 (13.3)	83 (20.8)	246 (61.5)	18 (4.5)	40 (10.0)

Abbreviation: FDA, US Food and Drug Administration.

^a^ Study indication was defined based on enrollment criteria and classified in relation to first FDA-approved indication.

^b^ Studies enrolling participants matching a supplemental FDA-approved indication, as identified in supplemental new drug applications.

#### Study Design Characteristics

Most of the 600 postapproval trials were nonrandomized (359 [59.8%]) with open-label allocation (455 [75.8%]). While 381 trials (63.5%) used no comparator, 117 trials (19.5%) were placebo-controlled, and 102 trials (17.0%) had active comparators, of which 13 (2.2%) actually evaluated the therapeutic of interest as an active comparator with another intervention. All of the 63 postapproval trials of therapeutics that received accelerated approval were open label. Median (IQR) enrollment and study duration were 44 (21-131) participants and 37 (22-57) months, respectively (Table 3).

**Table 3.** Study Design Characteristics for Postapproval Clinical Trials of Therapeutics Approved Without Postmarketing Requirements or Postmarketing Commitments for New Clinical Studies by the US Food and Drug Administration, 2009-2012

Therapeutic Characteristic	No. of Trials	No. (%)											Median (IQR)	
		Randomized Allocation	Blinding			Comparator			End Point^a^					
			Double	Single	Open label	Active^b^	Placebo	None^c^	Clinical Outcome	Clinical Scale	Surrogate Marker	Safety Outcome	Estimated or Actual Enrollment, No.	Study Duration, mo
Total	600	241 (40.2)	135 (22.5)	10 (1.7)	455 (75.8)	102 (17.0)	117 (19.5)	381 (63.5)	311 (51.8)	43 (7.2)	226 (37.7)	20 (3.3)	44 (21-131)	37 (22-57)
Class														
Drug	503	202 (40.2)	108 (21.5)	10 (2.0)	385 (76.5)	92 (18.3)	89 (17.7)	322 (64.0)	260 (51.7)	33 (6.6)	191 (38.0)	19 (3.8)	43 (22-123)	38 (23-58)
Biologic	97	39 (40.2)	27 (27.8)	0	70 (72.2)	10 (10.3)	28 (28.9)	59 (60.8)	51 (52.6)	10 (10.3)	35 (36.1)	1 (1.0)	48 (16-200)	29 (17-56)
Therapeutic area														
Cancer and hematology	437	115 (26.3)	43 (9.8)	1 (0.2)	393 (89.9)	44 (10.1)	56 (12.8)	337 (77.1)	200 (45.8)	20 (4.6)	199 (45.5)	18 (4.1)	40 (20-76)	43 (29-66)
Cardiovascular, diabetes, and hyperlipidemia	62	55 (88.7)	30 (48.4)	4 (6.5)	28 (45.2)	33 (53.2)	16 (25.8)	13 (21.0)	49 (79.0)	1 (1.6)	11 (17.7)	1 (1.6)	175 (47-678)	27 (18-47)
Autoimmune, musculoskeletal, and dermatology	33	18 (54.5)	15 (45.5)	2 (6.1)	16 (48.5)	0	18 (54.5)	15 (45.5)	20 (60.6)	10 (30.3)	2 (6.1)	1 (3.0)	76 (20-348)	13 (7-20)
Other	68	53 (77.9)	47 (69.1)	3 (4.4)	18 (26.5)	25 (36.8)	27 (39.7)	16 (23.5)	42 (61.8)	12 (17.6)	14 (20.6)	0	70 (24-230)	17 (8-26)
Priority review														
Yes	352	132 (37.5)	64 (18.2)	2 (0.6)	286 (81.3)	57 (16.2)	60 (17.0)	235 (66.8)	180 (51.1)	24 (6.8)	140 (39.8)	8 (2.3)	46 (24-121)	41 (25-59)
No	248	109 (44.0)	71 (28.6)	8 (3.2)	169 (68.1)	45 (18.1)	57 (23.0)	146 (58.9)	131 (52.8)	19 (7.7)	86 (34.7)	12 (4.8)	40 (16-149)	29 (16-52)
Accelerated approval														
Yes	63	11 (17.5)	0	0	63 (100.0)	5 (7.9)	3 (4.8)	55 (87.3)	33 (52.4)	0	29 (46.0)	1 (1.6)	42 (22-66)	49 (33-75)
No	537	230 (42.8)	135 (25.1)	10 (1.9)	392 (73.0)	97 (18.1)	114 (21.2)	326 (60.7)	278 (51.8)	43 (8.0)	197 (36.7)	19 (3.5)	45 (21-134)	36 (20-54)
Orphan status														
Yes	200	72 (36.0)	54 (27.0)	0	146 (73.0)	27 (13.5)	45 (22.5)	128 (64.0)	101 (50.5)	22 (11.0)	72 (36.0)	5 (2.5)	49 (22-139)	34 (19-55)
No	400	169 (42.3)	81 (20.3)	10 (2.5)	309 (77.3)	75 (18.8)	72 (18.0)	253 (63.3)	210 (52.5)	21 (5.3)	154 (38.5)	15 (3.8)	42 (20-128)	39 (23-58)

Abbreviation: IQR, interquartile range.

^a^ Trial end points were classified based on the highest level of efficacy evidence generated by primary or secondary outcome measures. From highest to lowest: clinical outcomes, clinical scales, and surrogate markers. *Safety outcome* refers to trials that assessed a safety outcome but no efficacy outcome.

^b^ Active comparator trials are those in which the therapeutic of interest was either compared with at least 1 active agent or was used as the active comparator with another therapeutic.

^c^ Clinical trials without a comparator include trials using single-group assignment, trials comparing the therapeutic of interest with observation (ie, no treatment), and trials in which all participants received the therapeutic of interest as part of various combination therapies.

Approximately half of postapproval trials (311 [51.8%]) assessed at least 1 clinical outcome among all primary and secondary end points, 85 (14.2%) of which were primary end points (Table 3). There were 43 trials (7.2%) that assessed clinical scales and 226 trials (37.7%) that used surrogate markers. While 20 trials (3.3%) assessed safety and not efficacy end points, 323 trials (53.8%) evaluated safety through at least 1 primary or secondary outcome measure. There were 86 trials (14.3%) with only pharmacokinetic, pharmacodynamic, dosing, or adherence measures as primary outcomes, but these evaluated safety or efficacy through secondary end points.

#### Status and Results Reporting on ClinicalTrials.gov

**Figure 2.**
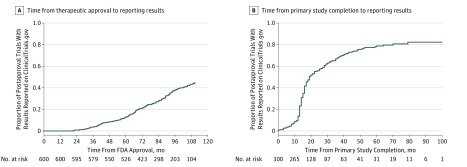
Time to Results Reporting on ClinicalTrials.gov for Postapproval Clinical Trials A, Time from original US Food and Drug Administration approval to reporting for all trials. B, Time from primary completion to reporting for completed or terminated trials.

As of July 2018, 218 trials (36.3%) were classified as completed on ClinicalTrials.gov, 82 trials (13.7%) were terminated, and nearly one-quarter (138 [23.0%]) were active but not recruiting participants (eTable 4 in the Supplement). Of the 300 completed or terminated postapproval trials for which results reporting on ClinicalTrials.gov would be expected within 12 months of primary completion, 204 (68.0%) had reported results (Figure 2) a median (IQR) 16 (13-25) months after their primary completion date and a median (IQR) 67 (50-84) months after first FDA approval of the studied therapeutic. For 177 of 204 trials with results (86.8%), results were first reported 12 months or longer after primary completion. Of the 300 completed or terminated trials, 96 trials (32.0%) did not have results on ClinicalTrials.gov, and the median (IQR) time since primary study completion was 35 (13-62) months.

## Discussion

We characterized the postapproval clinical evidence generated by trials sponsored by pharmaceutical companies for therapeutics first approved by the FDA from 2009 through 2012 without postmarketing requirements or commitments for new clinical studies and found variation in the number of subsequent trials conducted as well as in their purpose, quality, and timeliness. Although most therapeutics had at least 1 postapproval trial generating new safety or efficacy evidence, only 12% of trials were exclusively for the first FDA-approved indication. Instead, most trial investigated new indications or expanded patient populations. Most trials were nonrandomized, unblinded, and uncontrolled, and more than one-third focused on a surrogate marker of efficacy rather than a clinical end point. While 50% of postapproval trials were classified as completed or terminated, more than 85% of trials that reported results on ClinicalTrials.gov did so later than 1 year after their primary completion date.

Although pharmaceutical companies, which include approved manufacturers and associated collaborators, frequently conducted clinical trials after approval, even for therapeutics approved without clinical postmarketing requirements or commitments, more than 60% of such postapproval trials in this study were for new indications, and more than one-third of therapeutics had no postapproval trials exclusively studying the first FDA-approved indication. When postapproval trials are not required or voluntarily conducted, patients and clinicians are left to rely solely on the pivotal trials that supported FDA approval to inform treatment and prescribing decisions. However, pivotal trials vary in quantity, duration, and quality of end points assessed, particularly across clinical indications.^7^ Our findings are consistent with previous studies suggesting that therapeutics approved on the basis of limited evidence, such as single pivotal trials or surrogate markers of disease, generally do not have postapproval trials evaluating the same indications to assess clinical benefit.^15,17,18,19^ Instead, many trials evaluate unapproved uses as part of the development of supplemental indications for therapeutics,^18,29^ which may also drive off-label prescribing despite a lack of confirmatory evidence.^30^ Indeed, we found that more than 1 in 10 trials evaluated uses that at some point received FDA approval as supplemental indications, expanding the eligible treatment population. This pattern in postapproval trials has previously been observed for therapeutics granted accelerated approval^18^ and reinforces concerns that therapeutics may be rapidly integrated into standard treatment despite shortcomings in the available evidence.^30,31^

Although 68% of completed or terminated trials had results on ClinicalTrials.gov, most reported results later than 1 year after primary completion. The FDAAA requires that most clinical trials of FDA-approved therapeutics submit results to ClinicalTrials.gov within 1 year of their primary completion date.^28,32^ However, as of our download of ClinicalTrials.gov data on July 10, 2018, one-third of the completed or terminated postapproval trials in our sample remained without results reported for a median 35 months after completion or termination. This delay in evidence dissemination agrees with the findings of 2 studies^13,14^ on trials conducted to satisfy postmarketing requirements and commitments, both of which found results being reported on ClinicalTrials.gov years after their original FDA deadlines. Among therapeutics granted accelerated approval, a 2017 study^19^ suggested that only half of required confirmatory studies were completed at least 3 years after approval. These findings highlight the fact that both voluntary and required studies investigating approved therapeutic indications are frequently delayed. There are multiple potential causes of reporting delays, including difficulties in recruitment, extensions agreed on by the FDA, and nonadherence to reporting requirements on ClinicalTrials.gov. However, these causes are difficult to consistently determine using publicly available data. Regardless of the cause, such delays lead to clinical and regulatory questions remaining unanswered for years after approval.

As the party most invested in the development and marketing of therapeutics, pharmaceutical companies are uniquely positioned to conduct clinical trials that inform both life-cycle evaluation and patient care. However, required and voluntary postapproval trials for approved uses may be disincentivized by cost as well as the risk of identifying adverse events, moderating previous efficacy findings, or creating the perception that products have not been comprehensively evaluated.^21^ In the absence of postapproval trials, clinicians and regulators may rely on real-world sources of clinical data, such as electronic health records or the FDA Sentinel Initiative, which can help address questions regarding safety and efficacy. However, nonrandomized and unblinded studies do not always allow for causal interpretations and should be used only as a complement to clinical trial evidence.^6^ To ensure that patients and physicians have access to the highest-quality clinical evidence on approved indications, the FDA can outline more postmarketing requirements both at approval and during the market lives of therapeutics as new clinical questions arise. The FDA may also communicate evolving clinical and regulatory priorities to pharmaceutical companies, facilitating the development of postmarketing commitments. These efforts may become increasingly critical as expedited approval pathways continue to be used,^5^ resulting in more therapeutics reaching the market with limited evidence. If pharmaceutical companies do not voluntarily conduct postapproval trials that address persistent gaps in clinical knowledge on approved therapeutics, greater reliance on postmarketing requirements and commitments may be needed.^33^ Simultaneously, opportunities exist for pharmaceutical companies to improve timely reporting of results for voluntary and required postapproval trials, and despite reporting guidelines outlined in the FDAAA, more robust oversight of ClinicalTrials.gov may be needed. Future studies evaluating changes in the patterns of evidence generation from postmarketing requirements, postmarketing commitments, and voluntary postapproval studies may be necessary to determine whether improvements are being made over time.

### Limitations

Our study has several limitations. First, we only considered postapproval clinical trials sponsored or collaborated on by pharmaceutical companies, which include approved manufacturers and associated collaborators. Although clinical trials sponsored or collaborated on by pharmaceutical companies are also a source of postapproval safety and efficacy data,^17,34^ evidence suggests that these trials are often smaller in size and impact. Second, by searching for postapproval clinical trials registered on ClinicalTrials.gov, we may have missed unregistered trials or trials registered on other registries.^35^ However, US statute requires trials for FDA-approved therapeutics to be registered and their results reported on ClinicalTrials.gov,^28^ which remains an important public resource for US-based clinical research for patients and physicians.^26,27,36^ Third, we relied on ClinicalTrials.gov registration entries, which vary in the level of detail provided about trial indications and design. Fourth, our study focused on therapeutics first approved without postmarketing requirements or commitments for new clinical studies and did not account for required clinical studies that were ongoing at the time of approval. Additionally, our analyses did not account for potential differences in study objective and design between clinical trial phases (eg, phase 1 trials primarily intended to identify adverse events that also collected preliminary efficacy data). However, we sought to characterize all postapproval trials generating safety or efficacy evidence, regardless of primary trial objective.

## Conclusions

Postapproval clinical trials by pharmaceutical companies for novel therapeutics approved by the FDA from 2009 through 2012 without clinical postmarketing requirements or commitments varied in quantity, objective, and study design. While most therapeutics had at least 1 postapproval trial evaluating safety or efficacy, only 12% of trials exclusively studied first FDA-approved indications, and more than 60% of trials instead focused on unapproved or supplemental indications. Results reporting on ClinicalTrials.gov was frequently delayed. To ensure that patients and physicians have access to comprehensive clinical trial data informing treatment and prescribing decisions, robust communication between the FDA and pharmaceutical companies, including the effective use of postmarketing requirements and commitments, is essential to the investigation of therapeutic safety and efficacy after market approval.

## Notes

Supplement. **eAppendix 1.** Additional Methods Regarding Categorization of Drugs and Biologics Receiving Food and Drug Administration Approval for Their First Indication From 2009 Through 2012 Without Postmarketing Requirements or Postmarketing Commitments for New Clinical Studies **eTable 1.** Novel Therapeutics Approved From 2009 Through 2012 Without Postmarketing Requirements or Postmarketing Commitments for New Clinical Studies **eAppendix 2.** Additional Methods Regarding Identification of Postapproval Clinical Trials of Therapeutics Receiving Food and Drug Administration Approval for Their First Indication From 2009 Through 2012 Without Postmarketing Requirements or Postmarketing Commitments for New Clinical Studies **eTable 2.** Duplicate Clinical Trial Entries **eAppendix 3.** Additional Methods for Abstraction of Trial Data for Postapproval Clinical Trials of Therapeutics Receiving Food and Drug Administration Approval for Their First Indication From 2009 Through 2012 Without Postmarketing Requirements or Postmarketing Commitments for New Clinical Studies **eTable 3.** Trial Indication Classification **eTable 4.** ClinicalTrials.gov Status for Postapproval Clinical Trials of Therapeutics Approved by the Food and Drug Administration From 2009 Through 2012 Without Postmarketing Requirements or Postmarketing Commitments for New Clinical Studies **eReferences.**
