# Supplementary material for: Analysis of Postapproval Clinical Trials of Therapeutics Approved by the US Food and Drug Administration Without Clinical Postmarketing Requirements or Commitments
Source: JAMA Netw Open. 2019 May 10;2(5):e193410. doi: 10.1001/jamanetworkopen.2019.3410 (PMC6512282; doi:10.1001/jamanetworkopen.2019.3410)
Supplement: Supplement. — eAppendix 1. Additional Methods Regarding Categorization of Drugs and Biologics Receiving Food and Drug Administration Approval for Their First Indication From 2009 Through 2012 Without Postmarketing Requirements or Postmarketing Commitments for New Clinical Studies eTable 1. Novel Therapeutics Approved From 2009 Through 2012 Without Postmarketing Requirements or Postmarketing Commitments for New Clinical Studies eAppendix 2. Additional Methods Regarding Identification of Postapproval Clinical Trials of Therapeutics Receiving Food and Drug Administration Approval for Their First Indication From 2009 Through 2012 Without Postmarketing Requirements or Postmarketing Commitments for New Clinical Studies eTable 2. Duplicate Clinical Trial Entries eAppendix 3. Additional Methods for Abstraction of Trial Data for Postapproval Clinical Trials of Therapeutics Receiving Food and Drug Administration Approval for Their First Indication From 2009 Through 2012 Without Postmarketing Requirements or Postmarketing Commitments for New Clinical Studies eTable 3. Trial Indication Classification eTable 4. ClinicalTrials.gov Status for Postapproval Clinical Trials of Therapeutics Approved by the Food and Drug Administration From 2009 Through 2012 Without Postmarketing Requirements or Postmarketing Commitments for New Clinical Studies eReferences. [file jamanetwopen-2-e193410-s001.pdf]

Supplementary Online Content

Skydel JJ, Luxkaranayagam AT, Dhruva SS, Ross JS, Wallach JD. Analysis of postapproval clinical trials of therapeutics approved by the US Food and Drug Administration without clinical postmarketing requirements or commitments. *JAMA Netw Open*. 2019;2(5):e193410. doi:10.1001/jamanetworkopen.2019.3410

eAppendix 1. Additional Methods Regarding Categorization of Drugs and Biologics Receiving Food and Drug Administration Approval for Their First Indication From 2009 Through 2012 Without Postmarketing Requirements or Postmarketing Commitments for New Clinical Studies
eTable 1. Novel Therapeutics Approved From 2009 Through 2012 Without Postmarketing Requirements or Postmarketing Commitments for New Clinical Studies
eAppendix 2. Additional Methods Regarding Identification of Postapproval Clinical Trials of Therapeutics Receiving Food and Drug Administration Approval for Their First Indication From 2009 Through 2012 Without Postmarketing Requirements or Postmarketing Commitments for New Clinical Studies
eTable 2. Duplicate Clinical Trial Entries
eAppendix 3. Additional Methods for Abstraction of Trial Data for Postapproval Clinical Trials of Therapeutics Receiving Food and Drug Administration Approval for Their First Indication From 2009 Through 2012 Without Postmarketing Requirements or Postmarketing Commitments for New Clinical Studies
eTable 3. Trial Indication Classification
eTable 4. ClinicalTrials.gov Status for Postapproval Clinical Trials of Therapeutics Approved by the Food and Drug Administration From 2009 Through 2012 Without Postmarketing Requirements or Postmarketing Commitments for New Clinical Studies
eReferences.

This supplementary material has been provided by the authors to give readers additional information about their work.

© 2019 Skydel JJ et al. *JAMA Network Open.*

eAppendix 1. Additional Methods Regarding Categorization of Drugs and Biologics Receiving Food and Drug Administration Approval for Their First Indication From 2009 Through 2012 Without Postmarketing Requirements or Postmarketing Commitments for New Clinical Studies

Two investigators (JJS and JDW) confirmed the absence of postmarketing requirements and postmarketing commitments for new clinical studies, including new prospective cohort studies, registries, or clinical trials, for novel therapeutics approved by the FDA between 2009 and 2012 (**eTable 1**), using a previously described approach.[1]

Postmarketing requirement (PMR) status: Trials were classified based on postmarketing requirement status.

- "No Clinical PMR": Therapeutics approved by the FDA with postmarketing requirements that did not include new prospective cohort studies, registries, or clinical trials. Requirements could include new animal or other studies, and/or the completion of ongoing studies.
- "No PMR": Therapeutics approved by the FDA with no postmarketing requirements whatsoever.

No drugs or biologics were approved in this timeframe with postmarketing commitments but not postmarketing requirements for new clinical studies.

© 2019 Skydel JJ et al. *JAMA Network Open.*

**eTable 1.** Novel Therapeutics Approved From 2009 Through 2012 Without Postmarketing Requirements or Postmarketing Commitments for New Clinical Studies		
**Drug name (Brand name)**	**FDA-approved indication(s) at first approval**	**PMR status**
Abiraterone acetate (Zytiga)	Metastatic castration-resistant prostate cancer after receiving prior chemotherapy containing docetaxel	No Clinical PMR
Alcaftadine (Lastacaft)	Itching associated with allergic conjunctivitis	No PMR
Apixaban (Eliquis)	Prevention of stroke and systemic embolism in patients with non-valvular atrial fibrillation	No PMR
Asparaginase *Erwinia chrysathemi* (Erwinaze)	Patients with acute lymphoblastic leukemia (ALL) who have developed hypersensitivity to *E. coli*-derived asparaginase	No Clinical PMR
Axitinib (Inlyta)	Renal cell carcinoma after failure of one prior systemic therapy	No PMR
Benzyl alcohol (Ulesfia)	Head lice infestation in patients 6 months of age or older	No PMR
Bepotastine besilate (Bepreve)	Ocular itching associated with allergic conjunctivitis	No PMR
Bosutinib (Bosulif)	Chronic, accelerated, or blast phase Ph+ chronic myelogenous leukemia (CML) with resistance, or intolerance to prior therapy	No Clinical PMR
Canakinumab (Ilaris)	Cryopyrin-Associated Periodic Syndromes (CAPS)	No Clinical PMR
Clobazam (Onfi)	Seizures associated with Lennox-Gastaut Syndrome	No Clinical PMR
Collagenase *Clostridium histolyticum* (Xiaflex)	Dupuytren's contracture with a palpable cord	No Clinical PMR
Crizotinib (Xalkori)	Locally advanced or metastatic non-small cell lung cancer (NSCLC) that is anaplastic lymphoma kinase (ALK)-positive as detected by an FDA-approved test	No Clinical PMR
Dabigatran etexilate mesylate (Pradaxa)	Reduce the risk of stroke and systemic embolism in patients with non-valvular atrial fibrillation	No Clinical PMR
Dronedarone hydrochloride (Multaq)	Reduce the risk of cardiovascular hospitalization in patients with paroxysmal or persistent atrial fibrillation (AF) or atrial flutter (AFL), with a recent episode of AF/AFL and associated cardiovascular risk factors (i.e., age >70, hypertension, diabetes, prior cerebrovascular accident, left atrial diameter ≥50 mm or left ventricular ejection fraction [LVEF] <40%), who are in sinus rhythm or who will be cardioverted	No Clinical PMR
Eribulin mesylate (Halaven)	Metastatic breast cancer after having previously received at least two chemotherapeutic regimens	No Clinical PMR
Estradiol valerate and Estradiol valerate/Dienogest (Natazia)	Prevention of pregnancy	No Clinical PMR
Everolimus (Afinitor)	Advanced renal cell carcinoma after failure of treatment with sunitinib or sorafenib	No Clinical PMR
Glucarpidase (Voraxaze)	Toxic (> 1 micromole per liter) plasma methotrexate concentrations in patients with delayed methotrexate clearance due to impaired renal function.	No Clinical PMR
Icatibant acetate (Firazyr)	Acute attacks of hereditary angioedema	No Clinical PMR

© 2019 Skydel JJ et al. *JAMA Network Open.*

Indacaterol maleate (Arcapta Neohaler)	Long-term, once-daily maintenance bronchodilator treatment of airflow obstruction in patients with chronic obstructive pulmonary disease (COPD)	No PMR
Ingenol mebutate (Picato)	Actinic keratosis on face/scalp; actinic keratosis on trunk/extremities	No PMR
Ivacaftor (Kalydeco)	Cystic fibrosis in patients age 6 years and older who have a G551D mutation in the CFTR gene	No Clinical PMR
Lucinactant (Surfaxin)	Respiratory distress syndrome in premature infants	No PMR
Ofatumumab (Arzerra)	Chronic lymphocytic leukemia (CLL) refractory to alemtuzumab and fludarabine	No Clinical PMR
Pazopanib hydrochloride (Votrient)	Advanced renal cell carcinoma	No Clinical PMR
Pitavastatin (Livalo)	Primary hyperlipidemia and mixed dyslipidemia as an adjunctive therapy to diet to reduce elevated total cholesterol (TC), low-density lipoprotein cholesterol (LDL-C), apolipoprotein (Apo) B, and triglycerides (TG) and to increase high-density lipoprotein cholesterol (HDL-C)	No Clinical PMR
Polidocanol (Asclera)	Sclerose uncomplicated spider veins (varicose veins ≤1 mm in diameter) and uncomplicated reticular veins (varicose veins 1 to 3 mm in diameter) in the lower extremity	No Clinical PMR
Ponatinib (Iclusig)	Chronic phase, accelerated phase, or blast phase chronic myeloid leukemia (CML) that is resistant or intolerant to prior tyrosine kinase inhibitor therapy; Philadelphia chromosome positive acute lymphoblastic leukemia (Ph+ALL) that is resistant or intolerant to prior tyrosine kinase inhibitor therapy	No Clinical PMR
Prasugrel (Effient)	Reduction of thrombotic cardiovascular events (including stent thrombosis) in patients with acute coronary syndrome who are to be managed with percutaneous coronary intervention (PCI) as follows: Patients with unstable angina (UA) or non-ST-elevation myocardial infarction (NSTEMI), Patients with ST-elevation myocardial infarction (STEMI) when managed with either primary or delayed PCI	No Clinical PMR
Regorafenib (Stivarga)	Metastatic colorectal cancer (CRC) after previous treatment with fluoropyrimidine-, oxaliplatin- and irinotecan-based chemotherapy, an anti-VEGF therapy, and, if KRAS wild type, an anti-EGFR therapy	No Clinical PMR
Romidepsin (Istodax)	Cutaneous T-cell lymphoma (CTCL) in patients who have received at least one prior systemic therapy	No Clinical PMR
Ruxolitinib (Jakafi)	Intermediate or high-risk myelofibrosis, including primary myelofibrosis, postpolycythemia vera myelofibrosis and post-essential thrombocythemia myelofibrosis	No Clinical PMR
Tafluprost (Zioptan)	Reduction of intraocular pressure in patients with open angle glaucoma/ocular hypertension	No PMR
Ticagrelor (Brilinta)	Reduce the rate of thrombotic cardiovascular events in patients with acute coronary syndrome (ACS) (unstable angina, non-ST elevation myocardial infarction, or ST elevation myocardial infarction)	No PMR
Ulipristal acetate (Ella)	Prevention of pregnancy following unprotected intercourse or a known or suspected contraceptive failure	No Clinical PMR

© 2019 Skydel JJ et al. *JAMA Network Open.*

Velaglucerase alfa (Vpriv)	Gaucher disease (enzyme replacement therapy)	No PMR
Ziv-Aflibercept (Zaltrap)	Metastatic colorectal cancer resistant to or progressed following an oxaliplatin-containing regimen	No PMR

© 2019 Skydel JJ et al. *JAMA Network Open.*

eAppendix 2. Additional Methods Regarding Identification of Postapproval Clinical Trials of Therapeutics Receiving Food and Drug Administration Approval for Their First Indication From 2009 Through 2012 Without Postmarketing Requirements or Postmarketing Commitments for New Clinical Studies

One investigator (J.J.S.) identified postapproval clinical trials by searching ClinicalTrials.gov for generic and brand names of therapeutics using the “Intervention/Treatment” field, limiting the “Sponsors” filter to “Industry” and the “Study Start Dates” filter to one year prior to the original FDA approval date as listed on the Drugs@FDA database. Names were simplified when necessary to maximize returned search results (e.g. “abiraterone acetate” to “abiraterone”). When searches for the brand and generic names of therapeutics did not return the same number of hits, the broader results were used.

Two investigators (JJS and ATL) reviewed study entries downloaded from ClinicalTrials.gov on 10 July 2018. Ineligible studies were excluded based on information available in ClinicalTrials.gov entries. Eligibility criteria were applied sequentially as listed below, with any one criterion considered sufficient for exclusion. If a determination could not be made using available data (e.g., if no formulation was listed, no trial sites were provided, etc.), the study was not excluded on that criterion. In these cases, efforts were made to identify other sources of potential information, such as manufacturer websites or published clinical trial findings. All uncertainties were discussed between investigators (JJS, JSR, and JDW) and resolved by consensus.

Exclusion criteria:

- Studies that were completed prior to FDA approval date, as listed on the Drugs@FDA database.
- Studies that evaluated alternative FDA-approved or unapproved formulations of the active ingredient (e.g., formulations of everolimus that received separate FDA approval from Afinitor, everolimus-eluting stents, fixed-dose combinations lumacaftor/ivacaftor, etc.). When it could not be determined whether the therapeutic evaluated was the intended FDA-approved formulation, the trial was included unless study information such as dose strength, route of administration, study indication (in combination with FDA approval dates for approved therapeutic formulations), and/or an alternative brand names could be used to confirm that a different approved or unapproved formulation was being studied.
- Studies that provided therapeutics at the investigator’s discretion (e.g. “physician’s choice” of mTOR inhibitor) so that it could not be determined whether subjects received the therapeutic(s) of interest.
- Observational studies.
- Expanded access studies.
- Studies that did not enroll any new participants for that investigation, including but not limited to extension, rollover, follow-up, and sub-studies. Studies enrolling some, but not exclusively, new participants (e.g. enrolling new participants for a particular study arm or cohort) were included.
- Studies with sponsors and collaborators that did not include the drug manufacturer, a subsidiary, or a company involved in the development or marketing of the drug (e.g. a licensed co-marketer). For therapeutics subject to acquisition during development or marketing, all pre- and post-FDA approval license holders were included regardless of trial date. Google searches using combinations of the drug name, manufacturer name, and study sponsor/collaborator name were used to identify formal business and collaborative relationships between industry and study sponsors/collaborators.
- Studies conducted without study sites in the United States. International studies were included so long as they listed at least one site in the United States. When no site information was available on ClinicalTrials.gov, studies were included at the discretion of investigators based on available registration data, including enrollment criteria, sponsor data, and publications indexed to ClinicalTrials.gov entries.
- Studies that enrolled only healthy subjects.
- Studies that did not evaluate any safety or efficacy endpoints (e.g. PK/PD studies). Endpoints related to dosing, such as determination of maximum tolerated dose and dose-limiting toxicity, were considered “PK/PD” endpoints.

A total of 6 eligible clinical trials investigated more than one therapeutic in our sample as an intervention/treatment, resulting in duplicate study entries (**eTable 2**). For these trials, one entry was chosen at random for inclusion in all subsequent analyses.

© 2019 Skydel JJ et al. *JAMA Network Open.*

**eTable 2.** Duplicate Clinical Trial Entries	
**ClinicalTrials.gov identifier**	**Intervention/treatment therapeutics[a]**
NCT01184326	**Everolimus,** pazopanib
NCT01217931	Everolimus, **pazopanib**
NCT01441388	Axitinib, **crizotinib**
NCT01999972	**Axitinib,** crizotinib
NCT02693535	Axitinib, bosutinib, **crizotinib,** regorafenib
NCT02782403	**Axitinib,** bosutinib
[a] Trial entries randomly chosen for inclusion are bolded.	

eAppendix 3. Additional Methods for Abstraction of Trial Data for Postapproval Clinical Trials of Therapeutics Receiving Food and Drug Administration Approval for Their First Indication From 2009 Through 2012 Without Postmarketing Requirements or Postmarketing Commitments for New Clinical Studies

All clinical trial data was abstracted by two investigators (JJS and ATL) from study entries on ClinicalTrials.gov. Study design characteristics were defined based on ClinicalTrials.gov terminology.[2] Trial information was classified as follows:

Trial indication: Trial indications were defined using data available on ClinicalTrials.gov, including the "condition or disease" listed in the study description as well as eligibility criteria for enrollment. Trial indications were then classified by comparing to the first FDA approved indication(s) outlined in the original approval letter for the therapeutic of interest (**eTable 3**). Indications were classified narrowly based on the specific language of enrollment criteria (e.g. whether participants *must have* received vs. *may have* received previous therapy) and were confirmed as necessary using additional information, such as therapeutic labels available on the Drugs@FDA database or publications indexed to ClinicalTrials.gov entries or identified using NCT number.

Supplemental FDA-approved indication: Separate from trial indication classification, trials were classified as studying supplemental FDA-approved indications if a trial indication matched any additional FDA-approved indications outlined in a supplemental New Drug Application (sNDA) or the most recently-revised therapeutic label available for the therapeutic of interest on the Drugs@FDA database.

Allocation: Allocation to clinical trial arms was classified as "randomized" or "non-randomized" based on description of study design on ClinicalTrials.gov. "Randomized" trials were those explicitly described as such, while all other trials, including those using single group assignment, were classified as "non-randomized."

Masking: Trials were classified according to the level of blinding described on ClinicalTrials.gov.
- "Double blind": Trial was blinded, at minimum, to participant and investigator and/or outcomes assessor. In many cases, the trial was also blinded to care provider. Trials described as "triple" and "quadruple" blinded on ClinicalTrials.gov were reclassified as "double blind."
- "Single blind": Trial was blinded to participant, investigator, care provider, or outcomes assessor.
- "Open label": Trial was not blinded.

Comparator: Trials were classified according to the comparator or comparators used to evaluate the therapeutic of interest.
- "Active": The therapeutic of interest was compared to another active therapeutic or was given as the "active comparator" to another therapeutic being investigated.
- "Placebo": The therapeutic of interest was compared to a placebo.
- "None": The therapeutic of interest was not compared to another active agent or to a placebo comparator. This includes trials comparing the therapeutic of interest to observation (i.e. no treatment), single group assignment trials giving the therapeutic to all participants, or multi-arm trials in which all arms receive the therapeutic of interest at different doses or as a part of various combination therapies.

Endpoint: Primary and secondary outcome measures were classified based on a previously described approach.[3] Trials were then classified based on the highest level of efficacy evidence generated by a primary or secondary outcome measure ("clinical outcome," then "clinical scale," then "surrogate marker"). Trials evaluating safety but not efficacy endpoints were classified as "safety outcome." Trials were included if they evaluated at least one safety or efficacy endpoint as a primary or secondary outcome measure.
- "Clinical outcome": Endpoint directly assesses participant survival or function (e.g. overall survival).
- "Clinical scale": Endpoint assesses disease severity or patient symptoms using a graded scale or index (e.g. pain assessed on a visual analog scale).
- "Surrogate marker": Endpoint assesses expected clinical benefit using biomarkers (e.g. tumor response classified using Response Evaluation Criteria in Solid Tumor, RECIST version 1.1).
- "Safety outcome": Endpoint assesses tolerability or the incidence of adverse events.

© 2019 Skydel JJ et al. *JAMA Network Open.*

- “Other”: Endpoint assesses an outcome other than safety or efficacy, including pharmacokinetic or pharmacodynamic measures, drug-drug interactions, maximum tolerated dose, dose-limiting toxicity, utilization, or adherence (e.g. maximum observed plasma concentration, number of participants completing treatment, etc.).

Enrollment: The number of estimated (target) trial participants for trials open to enrollment, or the actual number of participants for clinical trials closed to enrollment, as provided by ClinicalTrials.gov study entry at the time of abstraction.

Study duration: The estimated study duration was calculated as the length of time, in months, between a trial’s “study start date” and its “primary completion date” as listed on ClinicalTrials.gov.

Time to results reporting: The time to results reporting was calculated as the length of time, in months, between a trial’s “primary completion date” and its “results first posted” date as listed on ClinicalTrials.gov. In addition, the length of time, in months, between the original FDA approval date for the evaluated therapeutic and a trial’s “results first posted” date was calculated. These values were used, respectively, as estimates of the delay between therapeutic approval and clinical evidence generation, and trial completion and clinical evidence generation.

ClinicalTrials.gov status: Trials were classified as “not yet recruiting,” “recruiting,” “enrolling by invitation,” “withdrawn,” “active, not recruiting,” “suspended,” “terminated,” “completed,” or “unknown status,” based on the most recent recruitment status available on ClinicalTrials.gov (**eTable 4**). “Completed” and “terminated” trials have ceased to treat or examine participants and in most cases are expected to submit basic results within one year of primary completion date.[4] “Active, not recruiting” trials are ongoing but may begin to post study results prior to completion or termination.

© 2019 Skydel JJ et al. *JAMA Network Open.*

**eTable 3.** Trial Indication Classification	
First FDA-approved	A trial evaluating a therapeutic for an indication found in the initial FDA approval letter, meeting the specific requirements of that indication, including individual patient characteristics (e.g., BCR-ABL positive, previous sorafenib treatment, etc.).
Modified first FDA-approved	A trial evaluating a therapeutic for its first indicated disease, but in expanded patient populations (e.g. pediatric use when originally approved for adults, as first-line rather than second-line therapy, etc.).
FDA-unapproved	A trial evaluating a therapeutic for an indication not approved by the FDA at the time of first approval.
Multiple indications	A trial evaluating a therapeutic for more than one indication, potentially including first FDA-approved, modified first FDA-approved, and/or FDA-unapproved indications.

© 2019 Skydel JJ et al. *JAMA Network Open.*

eTable 4. ClinicalTrials.gov Status for Postapproval Clinical Trials of Therapeutics Approved by the Food and Drug Administration From 2009 Through 2012 Without Postmarketing Requirements or Postmarketing Commitments for New Clinical Studies

ClinicalTrials.gov status	No. (%) of studies	
	Clinical trials	Results reported on ClinicalTrials.gov
Not yet recruiting, recruiting, or enrolling by invitation	124	0 (0.0)
Withdrawn	30	0 (0.0)
Active, not recruiting	138	7 (5.1)
Suspended	2	0 (0.0)
Terminated	82	62 (75.6)
Completed	218	142 (65.1)
Unknown status	6	0 (0.0)
Total	600	211 (35.2)

© 2019 Skydel JJ et al. *JAMA Network Open.*

eReferences.

1. Wallach JD, Egilman AC, Dhruva SS, et al. Postmarket studies required by the US Food and Drug Administration for new drugs and biologics approved between 2009 and 2012: a cross-sectional evaluation. *BMJ*. 2018;361:k2031. doi: 10.1136/bmj.k2031
2. NIH: US National Library of Medicine. Glossary of common site terms – ClinicalTrials.gov. ClinicalTrials.gov. https://clinicaltrials.gov/ct2/about-studies/glossary/. Published November 2018. Accessed December 2, 2018.
3. Downing NS, Aminawung JA, Shah ND, Krumholz HM, Ross JS. Clinical trial evidence supporting FDA approval of novel therapeutic agents, 2005-2012. *JAMA*. 2014;311(4):368-377. doi:10.1001/jama.2013.282034
4. NIH: US National Library of Medicine. FDAAA 801 and the Final Rule - ClinicalTrials.gov. ClinicalTrials.gov. https://clinicaltrials.gov/ct2/manage-recs/fdaaa/. Published April 2018. Accessed October 22, 2018.

© 2019 Skydel JJ et al. *JAMA Network Open.*
